# Understanding the impact of glycolysis and oxidative stress on residual feed intake in slow-growing chicken

**DOI:** 10.1016/j.psj.2025.105816

**Published:** 2025-09-10

**Authors:** Rattanaporn Niyomphong, Pramin Kaewsatuan, Saknarin Pengsanthia, Wittawat Molee, Amonrat Molee

**Affiliations:** School of Animal Technology and Innovation, Institute of Agricultural Technology, Suranaree University of Technology, Nakhon Ratchasima 30000, Thailand

**Keywords:** Korat chicken, Feed efficiency, Slow-growing chicken, Glycolysis, Oxidative stress

## Abstract

Improving feed efficiency (FE) in slow-growing Korat chickens (KRC) is crucial for enhancing productivity and reducing production costs for sustainability. This study aimed to investigate the expression of genes and proteins involved in glycolysis and oxidative stress pathways and to identify potential metabolic biomarkers associated with residual feed intake (RFI). A total of 30 male KRCs were selected based on extreme RFI values at 10 weeks of age, forming low-RFI and high-RFI groups (n = 15/group). Duodenal samples were collected to extract total RNA and protein. Gene expression levels of *Aldolase C* (*ALDOC*), *Lactate Dehydrogenase B* (*LDHB*), *Phosphoglycerate Mutase 1* (*PGAM1*), *Triphosphate Isomerase* (*TPI1*), *Superoxide Dismutase 1* (*SOD1*), and *Peroxiredoxin-1* (*PRDX1*) were analyzed using real-time quantitative PCR, while protein levels of LDHB and SOD1 were analyzed by Western blot. Plasma glucose, pyruvate, and protein carbonyl content were also measured. Low-RFI chickens exhibited significantly higher mRNA and protein expression of LDHB, as well as elevated TPI1 mRNA levels (P < 0.05). Conversely, HRFI chickens showed increased expression of SOD1 at both mRNA and protein levels, along with significantly higher plasma glucose and protein carbonyls and lower pyruvate concentrations (P < 0.05). Principal Component Analysis (PCA) revealed a clear separation between RFI groups, with the *LDHB* gene associated with the low-RFI group. Whereas the *SOD1* gene and SOD1 protein, along with plasma glucose and protein carbonyl content, were positively correlated with the high-RFI group. These findings suggest that *LDHB* and *SOD1* genes may serve as potential biomarkers for FE, reflecting differences in energy metabolism and oxidative stress response in slow-growing chickens. This highlights the potential application of these markers in genetic selection and nutritional strategies to improve FE in poultry.

## Introduction

Chicken farming is a cornerstone of economic stability and livelihood for smallholder farmers across Southeast Asia, providing vital employment opportunities (Birhanu et al., 2023; Madilindi et al., 2023). However, poultry production, particularly feed manufacturing and farming practices, significantly contributes to nitrogen emissions and environmental pollution (Malomo et al., 2018; Wongtangtintharn et al., 2025). Consequently, feed efficiency (FE) stands as a crucial selection criterion in breeding programs, profoundly influencing the economic viability of animal protein production (Marchesi et al., 2021; Kaitholil et al., 2023). Residual feed intake (RFI), defined as the difference between actual and expected feed intake adjusted for body size and performance (Koch, 1963), serves as an effective indicator of individual animal FE. Low-RFI animals are advantageous as they reduce emissions and waste (Zhang et al., 2017) while maintaining normal growth and production traits (Alende et al., 2016; Bonilha et al., 2017; Zhang et al., 2019). Thus, identifying the genetic and molecular underpinnings of FE is paramount for developing sustainable and economically viable poultry breeding strategies.

Recent advancements in high-throughput technologies, such as transcriptomics, proteomics, and metabolomics, have significantly enhanced our understanding of the molecular mechanisms governing FE in livestock. Research has linked FE to molecular and physiological alterations across various tissues, including the intestine (Vigors et al., 2016; Sinpru et al., 2021; Kaewsatuan et al., 2022; Win et al., 2024), breast muscle (Grubbs et al., 2014; Vigors et al., 2019; Poompramun et al., 2021), and liver (Grubbs et al., 2014; Reyer et al., 2017; Vigors et al., 2019). The duodenum, central to digestion and nutrient absorption, is particularly relevant to FE research (Recoules et al., 2019). Previous duodenal transcriptomic studies, for instance, have highlighted key pathways influencing FE, such as metabolism, digestibility, energy homeostasis, and biosynthesis (Yi et al., 2015). Furthermore, high-FE individuals are often characterized by increased glucose uptake and higher glycolytic rates, typically associated with the overexpression of glycolysis-related genes and proteins like glucose transporter type 1 (*SLC2A1*), lactic dehydrogenase A (*LDHA*), and hexokinase (*HK1-HK3*) (Wang et al., 2019; Yang et al., 2020). Conversely, low-FE animals frequently exhibit elevated levels of protein carbonyls and antioxidant enzymes, indicating increased oxidative stress and potentially impaired detoxification of reactive oxygen species (ROS). This oxidative imbalance can disrupt energy metabolism, compromise intestinal integrity, and ultimately diminish feed utilization efficiency (Zhang, 2023). Our own previous proteomic analysis of the duodenum in slow-growing chickens with high- and low-FE further identified differentially abundant proteins (DAPs) significantly enriched in pathways related to glucose metabolism and oxidative stress, including aldolase C (ALDOC), lactate dehydrogenase B (LDHB), phosphoglycerate mutase 1 (PGAM1), triosephosphate isomerase (TPI1), superoxide dismutase 1 (SOD1), and peroxiredoxin-1 (PRDX1) (Kaewsatuan et al., 2022). While these findings collectively suggest that targeting key regulators of glucose metabolism and antioxidant defense could improve FE in slow-growing chickens, the precise regulatory origins of these observed molecular changes, whether transcriptional, post-translational, or upstream signaling, remain poorly understood. Addressing this knowledge gap is crucial for improving our understanding of FE and identifying reliable molecular markers for selective breeding.

Consequently, this study had two main objectives. First, we aimed to compare the expression levels of selected genes and proteins related to glycolysis and oxidative stress using quantitative real-time PCR (qPCR) and Western blotting, respectively, alongside key metabolic indicators—namely glucose, pyruvate, and protein carbonyls—between high- and low-RFI chickens. Second, we employed principal component analysis (PCA) to identify which of these molecular factors contributes most significantly to variations in FE. This research focuses on the Korat chicken (KRC), a crossbreed of Thai Indigenous Leung Hang Khao and the Suranaree University of Technology (SUT) synthetic line. KRC is highly valued in Thai local farming systems for its favorable carcass quality, heat tolerance, and disease resistance, serving as both a protein source and income generator for small- to medium-scale farmers (Katemala et al., 2023). Despite these merits, KRC productivity remains relatively low, underscoring the need for genetic improvement. Thus, the present study contributes novel molecular data on KRC, offering insights into mechanisms regulating FE, which can potentially support future marker-assisted selection strategies in poultry breeding.

## Material and method

### Ethics statement

The experiment was conducted at the experimental farm of the Suranaree University of Technology (SUT), Thailand. All animal protocols were approved by the Ethics Committee on Animal Use of the Suranaree University of Technology, Nakhon Ratchasima, Thailand (Approval ID: SUT-IACUC-001/2021).

### Experiment chickens and phenotypic data collection

A total of 115 one-day-old male KR chickens were sexed using the vent sexing method, wing-banded, and vaccinated against Marek’s disease. All birds were individually housed in cages with ad libitum access to feed and water under consistent environmental conditions. The same diet was provided to all birds throughout the experiment, with crude protein (CP) levels of 21% for starter chickens (0-3 weeks), 19% for growers (4-6 weeks), and 17% for finishers (7-10 weeks). Individual body weight and feed intake were recorded weekly from 1 to 10 weeks of age to calculate RFI according to the method described by Izadnia et al. (2019) using the following formula:

$$\mathrm{RFI}=\mathrm{FI}-\left(\alpha +\beta_{1}BW^{0.75}+\beta_{2}\Delta \mathrm{BW}\right)$$

where *FI* is the feed intake, *BW^0.75^* is the metabolic weight estimated from the mean body weight at week, *ΔBW* is the body weight gain (g) during the week, *α* is the intercept, and *β_1_* and *β_2_* are partial regression coefficients.

At 10 weeks of age, chickens were then ranked based on their RFI values and divided into two groups. The 15 birds with the highest RFI values (ranging from 143.094 to 339.851) and the 15 with the lowest RFI values (ranging from -292.779 to -138.003) were selected for sampling. These groups are hereafter referred to as the high-RFI and low-RFI groups, respectively. Significant differences in RFI values between the two groups were assessed using Student’s t-test, with a significance threshold of P < 0.05.

### Duodenal and blood sample collection

At the end of the experiment, the chickens were electrically stunned and exsanguinated after 8 h of fasting. Prior to slaughter, blood samples were collected from each selected chicken in both the low-RFI and high-RFI chicken groups. After 8-hr of fasting. The collected blood samples were lysed and centrifuged at 3,500 rpm for 10 minutes at 4°C. Plasma was carefully extracted, transferred to microcentrifuge tubes (approximately 1.5 mL per tube), and stored at −20°C for subsequent analysis of key molecules associated with the glycolysis pathway and oxidative stress. Specifically, glucose was measured using a glucose oxidase assay, pyruvate content was determined via a pyruvic acid content assay, and protein carbonyl content was assessed using a protein oxidation assay.

After the chickens were killed, the intestinal tract was promptly removed, and the whole duodenum was collected and snap-frozen in liquid nitrogen at −80°C until further analysis of gene and protein expression. Dissecting instruments were sterilized with 70% ethanol after processing each chicken to prevent cross-contamination.

### RNA extraction

Total RNA extraction was performed as previously described by Sinpru et al. (2021). Approximately 0.5 g of duodenal tissue from each chicken was lysed and homogenized in TRIzol reagent (Thermo Fisher Scientific, Waltham, MA, USA). After adding 200 µL of Chloroform-isoamyl alcohol, 24:1 (v/v), the samples were incubated for 5 min at 25°C. The samples were then centrifuged at 12,000 g for 10 min at 4°C (Thermo Fisher Scientific, Waltham, MA, USA), and the supernatants were transferred to new tubes, incubated with chloroform for 5 min at 4°C, and centrifuged. The total RNA was precipitated from the resulting supernatants using 50 µL of 3 M sodium acetate and 500 µL of isopropanol, followed by washing with 75% ethanol and air-drying for 10 minutes at 25°C. RNA concentration and purity were determined using a spectrophotometer (NanoDrop 2000, Thermo Fisher Scientific, Waltham, MA, USA) at an absorbance of 260 nm, and RNA integrity was assessed by 1% agarose gel electrophoresis, showing sharp 28s and 18s rRNA bands with no smear, confirming that the RNA was intact and suitable for gene expression analysis.

### Quantitative reverse transcription polymerase chain reaction analysis

The total RNA was reverse-transcribed into first-strand cDNA using the High-Capacity cDNA Reverse Transcription Kit (Invitrogen, Carlsbad, CA, USA) according to the manufacturer's guidelines. The synthesized cDNA was diluted 10-fold for use as a RT-qPCR template and stored at −20°C until analysis. The RT-qPCR reactions were carried out on a LightCycler 480 Real-Time PCR System (Roche, Mannheim, Germany) using the first-strand cDNA templates, specific primers, and the SYBR Green PCR Master Mix (Thermo Fisher Scientific, Carlsbad, CA, USA). The oligonucleotide primers for the following genes were listed in Table 1: aldolase C (*ALDOC*), lactate dehydrogenase B (*LDHB*), phosphoglycerate mutase 1 (*PGAM1*), triphosphate isomerase (*TPI1*), superoxide dismutase 1 (*SOD1*), and peroxiredoxin-1 (*PRDX1*). The Actin Beta (*ACTB*) was employed as an internal control used to normalize the expression level of target transcripts (Sinpru et al., 2021). The PCR efficiency test was performed using serial dilutions of the cDNA pool and primers. Amplification efficiency was determined from the slope of the standard curve, and only primers with efficiencies ranging between 90% and 100% with R² ≥ 0.99 were used in the RT-qPCR reaction. All reactions were performed in triplicate. The thermocycling program consisted of an initial denaturation step at 94°C for 30 s, followed by 40 cycles of 94°C for 30 s, 54°C for 30 s, and 72°C for 1 min, with a final extension at 72°C for 5 min. The average cycle threshold (Ct) values were determined for each sample, and relative gene expression was calculated using the comparative 2−^ΔΔ^CT method (Livak et al., 2001), with ACTB serving as the reference gene. Fold-change values were used to represent the relative quantification of each target gene (Sinpru et al., 2021).

**Table 1:** Primer sequences and their applications.

Name	Primer Sequence (5′ to 3′) Forward/reverse primers	Annealing Temp	PCR (bp)	Reference
*ACTB*	5′-TGACCGCGTTACTCCCACAG -3′ 5′-CGAAACCGGCCTTGCACATA-3′	54°C	90	GenBank accession no. NM_205518.1
*ALDOC*	5′-CTCAACGCCATCAACACA-3′ 5′-CACGCTTGACGAACTCCT-3′	54°C	145	Wang et al. (2007)
*PRDX1*	5′-GTACAGTGACAGAGCTGATGAA-3′ 5′-GCAAGGTGACAGAAGTGAGA-3′	54°C	84	Zhang et al. (2023)
*SOD1*	5′-AGGGGGTCATCCACTTCC-3′ 5′-CCCATTTGTGTTGTCTCCAA-3′	54°C	122	Mountzouris et al. (2020)
*LDHB*	5′-TTCCCAGCAACAAGATCACCGT-3′ 5′-AACACCTGCCACATTAACTCCG-3′	54°C	511	Imagawa et al. (2006)
*TPI1*	5′-CTTGCCTATGAGCCAGTTTG-3′ 5′-GTTCCTTACAGTTGCCACCA-3′	54°C	175	Teltathum and Mekchay. (2009)
*PGAM1*	5′-CCTGCCCTTCTGGAATGAG-3′ 5′-TGGGCTTCAGGTTCTTGTCC-3′	54°C	191	Teltathum and Mekchay. (2009)

*ACTB*, actin beta; *ALDOC*, aldolase C; *PRDX1,* peroxiredoxin-1; *SOD1*, superoxide dismutase 1; *LDHB*, lactate dehydrogenase; *TPI1*, triphosphate isomerase; and *PGAM1*, phosphoglycerate mutase 1.

### Protein oxidation

Protein oxidation was measured based on the method described by Katemala et al. (2018), with minor modifications. Two equal aliquots of plasma were prepared: one was treated with 0.2% (w/v) 2,4-dinitrophenylhydrazine (DNPH) dissolved in 2 M HCl, while the other was treated with 2 M HCl alone and served as the blank. Samples were centrifuged at 4,500 rpm for 5 minutes at 4°C. The resulting pellets were washed with 10% trichloroacetic acid (TCA) to remove unbound DNPH, followed by repeated washes with an ethanol:ethyl acetate mixture (1:1, v/v) until the pellets appeared clear. The pellets were then dried under a stream of nitrogen gas and subsequently dissolved in 20 mM sodium phosphate buffer (pH 6.5) containing 6 M guanidine hydrochloride. The carbonyl content was measured at 562 nm as protein hydrazones and expressed as nanomoles of carbonyl per milligram of protein. Bovine serum albumin was used as the protein standard.

### Sodium dodecyl-sulfate polyacrylamide gel electrophoresis (SDS-PAGE) and western blotting analysis

SDS-PAGE and Western blotting analyses were performed according to the method described by Zhao et al. (2020a), with minor modifications. Frozen duodenal tissues were homogenized in RIPA lysis buffer (MedChemExpress, USA) containing 50 mM Tris pH 7.4, 150 mM NaCl, 1% Triton X-100, 1% sodium deoxycholate, and 0.1% SDS. Protein concentrations were then determined using a BCA Protein Assay Kit (Thermo Fisher Scientific, Waltham, MA, USA). Ten micrograms of total protein per sample were separated on SDS-PAGE gels consisting of a 4% stacking gel and a 10% separating gel (Whatman Protran, Dassel, Germany). After electrophoresis, proteins were transferred onto PVDF membranes using a semi-dry electroblotting system (catalog no. 170–3940; Bio-Rad, Hercules, CA, USA) at 50 mA for 2 hours. Membranes were blocked overnight at 4°C in Tris-buffered saline containing 0.1% Tween 20 (TBST) and 5% skim milk. After blocking, the membranes were washed four times with TBST (20 minutes each), then incubated with primary antibodies for 4 hours at room temperature. The primary antibodies used were LDHB rabbit polyclonal antibody (catalog no. 14824-1-AP; 1:1,000 dilution) and SOD1 rabbit polyclonal antibody (catalog no. 10269-1-AP; 1:1,000 dilution), both obtained from Proteintech Group, Inc. (Rosemont, USA). These antibodies have been previously validated for use in chickens (Zhao et al., 2020b; Ayoubi et al., 2023). Following primary antibody incubation, membranes were washed four times with TBST and incubated for 2 hours at room temperature with an HRP-conjugated secondary antibody: Affinipure Goat Anti-Rabbit IgG (1:5,000 dilution; catalog no. SA00001-2, Proteintech Group, Inc., Rosemont, USA). Western blot signals were visualized using the Bio-Rad ChemiDoc MP Imaging System, and target protein levels (LDHB and SOD1) were normalized to β-tubulin rabbit polyclonal antibody (1:1000 dilution; catalog no. 10094-1-AP, Proteintech Group, Inc., Rosemont, USA) and expressed as relative band intensities. No difference in β-tubulin band intensities was observed between the RFI group (P = 0.1152), supporting its use as a loading control. Densitometric analysis of protein bands was performed using ImageJ software (NIH, Bethesda, MD, USA).

### Glucose oxidase method

Plasma samples and glucose standards were gently mixed and loaded into the Biosystems BA400/BA200 analyzer (BioSystems S.A., Barcelona, Spain). The assay utilized the glucose oxidase method, which involves an enzymatic reaction leading to the formation of a colored product, subsequently quantified by absorbance measurements at 505/670 nm. Plasma glucose levels were calculated using a standard calibration curve derived from the glucose standards.

### Determination of pyruvic acid content

Pyruvic acid content was measured using a Pyruvic Acid Assay Kit (catalog no. abx097982, Abbexa, TX, USA). Plasma samples and standards were added to 96-well plates according to the instructions, and reaction solutions were added and detected under the microplate reader absorbance at OD 520 nm, and recorded to calculate the pyruvate level in the samples according to the standard curve. We set up each standard and sample in triplicate.

### Significant difference analysis

The group student’s t-test was used to evaluate significant differences in mean values between low-RFI and high-RFI chicken groups for the following parameters: feed intake, body weight gain, residual feed intake, feed conversion ratio, gene expression levels of *ALDOC, LDHB, TPI1, PGAM1, SOD1, and PRDX1*; protein expression levels of LDHB and SOD1; and plasma concentrations of glucose, pyruvate, and protein carbonyls. A significance level of α = 0.05 was applied. All statistical analyses were conducted using SPSS software version 29 (IBM Corp., Armonk, NY, USA).

### Principal component analysis

Principal component analysis (PCA) was applied to cluster and explore the relationships among gene expression, protein levels, and key metabolic indicators associated with the glycolysis pathway and oxidative stress in extreme low-RFI and high-RFI chicken samples. The analysis was performed using Unscrambler X Multivariate Data Analysis software (version 10.1; Camo Analytics, Oslo, Norway). The data matrix was standardized using the standard deviation of all variables before performing PCA. A biplot was then generated to visualize the correlations between variables and the clustering patterns among samples.

## Results

### Feed efficiency and growth performance of low-RFI and high-RFI KR chickens

The mean values and standard errors of total feed intake, body weight gain, and residual feed intake of KR chickens are presented in Table 2. A significant difference was observed in total feed intake and RFI between the low-RFI and high-RFI groups at 10 weeks of age (P < 0.01), with higher values recorded in the high-RFI groups. In contrast, no significant difference in body weight gain was detected between the two groups (P > 0.05). Although growth performance was comparable, the low-RFI group consumed significantly less feed than the high-RFI group. This indicates that low-RFI groups are more feed-efficient, as they achieve similar weight gain with reduced feed intake.

**Table 2:** Feed intake accumulation and body weight gain from low-RFI and high-RFI KR chickens at 10 weeks of age (mean±standard error).

Traits	Low-RFI (n = 15)	High-RFI (n = 15)	*P* value^1^
FI (g)	1688.267 ± 44.457	1841.600 ± 40.378	<0.01
BWG (g)	1349.202 ± 20.945	1375.482 ± 20.495	0.658
RFI	-199.373 ± 13.790	200.763 ± 15.130	<0.01

FI, feed intake; BWG, body weight gain; RFI, residual feed intake (mean ±SE).

1 Comparison between Low-RFI and High-RFI chicken groups by a t-test.

### Expression of genes involved in glycolysis and oxidative stress pathway in Korat chickens with different RFI groups

**Fig. 1:**
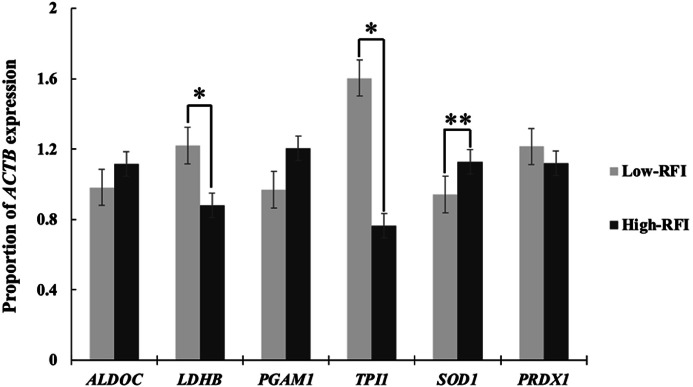
Quantitative real-time PCR of gene expression in duodenum chickens with high- and low-RFI in glycolysis and oxidative stress pathway. The histograms represent the mean value of gene expression level in the duodenum obtained from broilers with high- and low-RFI. Each bar represents the mean ± SE (n=15). *, *P* < 0.005 and **, *P* < 0.05. *ALDOC*, aldolase C; *LDHB*, lactate dehydrogenase; *PGAM1*, phosphoglycerate mutase 1; *TPI1*, triphosphate isomerase; *SOD1*, superoxide dismutase 1; *PRDX1*, peroxiredoxin-1.

The expression levels of genes involved in glycolysis and oxidative stress pathways in the duodenum of low-RFI and high-RFI groups are presented in Fig. 1. Among the glycolysis-related genes, lactate dehydrogenase B (*LDHB*) and triosephosphate isomerase 1 (*TPI1*) were significantly upregulated in the low-RFI group, with P-values of 0.004 and 0.001, respectively. In contrast, no significant differences were observed in the expression levels of aldolase C (*ALDOC*) and phosphoglycerate mutase 1 (*PGAM1*) between the two RFI groups. Regarding oxidative stress-related genes, superoxide dismutase 1 (*SOD1*) expression was significantly lower in low-RFI chickens compared to high-RFI groups (P < 0.05). However, the expression of peroxiredoxin-1 (*PRDX1*) did not differ significantly between the groups (P > 0.05). Although most genes related to glycolytic activity and oxidative stress regulation showed differential expression, the mRNA expression levels of *ALDOC, PGAM1*, and *PRDX1* did not appear to be strongly associated with the observed variations in FE between high-RFI and low-RFI groups, suggesting that differences in FE may be only partially attributed to these genes.

### Protein expression analysis for LDHB and SOD1 in the duodenum of chickens with low- and high-RFI using western blot technique

**Fig. 2:**
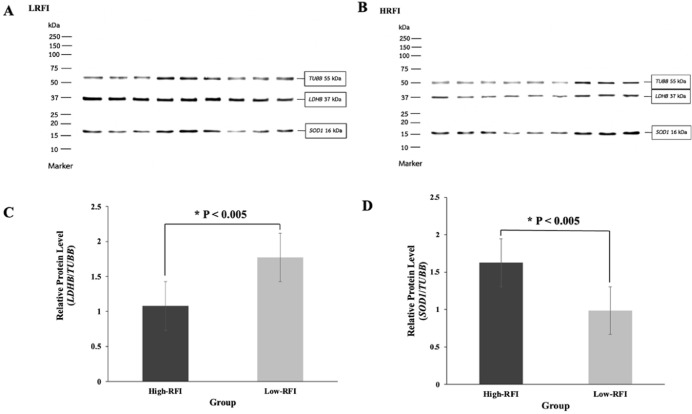
The effects of LDHB, SOD1, and TUBB (β-tubulin) protein expression associated with glycolysis and oxidative stress in the duodenum of Korat chickens with high- and low-RFI were analyzed using the Western blot technique. (A) Protein bands and LDHB, SOD1, and TUBB protein proportions in the low-RFI chicken group. (B) Protein bands and LDHB, SOD1, and TUBB protein proportions in the high-RFI chicken group. (C) Bar graph showing the density data for LDHB protein expression. (D) Bar graph showing the density data for SOD1 protein expression, standardized using TUBB (housekeeping protein), and analyzed with ImageJ software (NIH, Bethesda, MD, USA). Each bar represents the mean ± SE (n=15). *, P < 0.005.

The expression levels of LDHB and SOD1 proteins in the duodenum of chickens with low-RFI and high-RFI groups were determined using the Western blot technique, as illustrated in Fig. 2. Chickens with low-RFI groups exhibited significantly higher expression levels of the LDHB protein compared to those with high-RFI groups (P < 0.05; Fig. 2A and 2C). Additionally, low-RFI groups showed significantly lower expression levels of the SOD1 protein compared to high-RFI groups (P < 0.05; Fig. 2B and 2D). Thus, changes in the abundance of LDHB and SOD1 proteins may play a key role in determining differences in FE.

### Key metabolic indicators involved in glycolysis and oxidative stress pathway

**Fig. 3:**
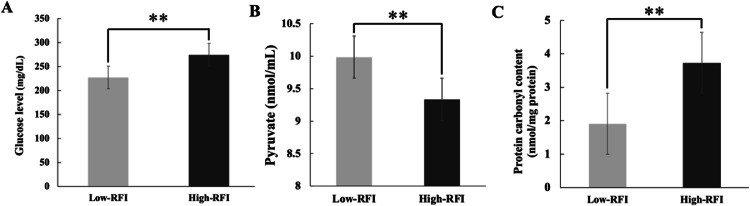
The key metabolic molecules involved in glycolysis and oxidative stress with high- and low-RFI chickens. (A) The histograms represent the mean of glucose level, (B) Relative amounts of pyruvate content obtained from chicken with high- and low-RFI, and (C) Protein carbonyl content in the duodenum obtained with high- and low-RFI chicken. Each bar represents the mean ± SE (n=15). **, P < 0.05.

Fig. 3 presents the levels of plasma metabolites (i.e., glucose, pyruvate, and protein carbonyl) in chickens with low-RFI and high-RFI groups. Significant differences were observed in glucose, pyruvate, and protein carbonyl levels between the low-RFI and high-RFI KR chicken groups (P < 0.05), suggesting that FE regulation may be influenced by these metabolic and oxidative stress-related markers.

### Principal component analysis and correlation loading of gene and protein expression and key metabolite levels of the glycolysis and oxidative stress pathways from low-RFI and high-RFI KR chickens

**Fig. 4:**
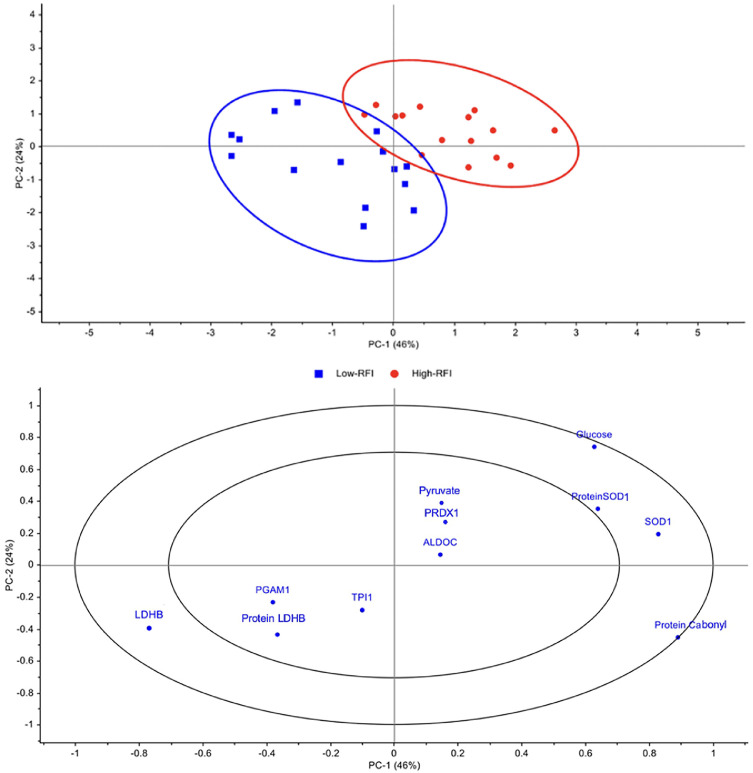
PCA score plot for PC1 versus PC2 from high-RFI and low-RFI data (upper) and correlation loading plot for PC1 versus PC2 for gene, protein expression, and metabolites involved with the glycolysis and oxidative stress pathway (lower). PCA, principal component analysis; PC, principal components; Low-RFI, low residual feed intake; High-RFI, high residual feed intake; *ALDOC*, aldolase C; *LDHB*, lactate dehydrogenase B gene; *PGAM1*, phosphoglycerate mutase 1 gene; *TPI1*, triphosphate isomerase gene; *SOD1*, superoxide dismutase 1 gene; *PRDX1*, peroxiredoxin-1 gene; ProteinSOD1, superoxide dismutase 1; Protein LDHB, lactate dehydrogenase B.

PCA was performed to determine which of the genes, proteins, or metabolic molecules related to glycolysis and oxidative stress contribute most to the variation in FE (Fig. 3). The score plot (Fig. 4, upper), focusing on PC1 (vertical axis), showed a clear separation between the two groups of chickens, accounting for 70 % of variance. In the loading plot (Fig. 4, lower), all variables located in the outer circle of the loading plot—specifically the *SOD1* gene, *LDHB* gene, SOD1 protein, glucose, and protein carbonyl—showed strong correlations with RFI variation. On the left side of the loading plot, the low-RFI groups were positively associated with *LDHB* gene expression, whereas the *SOD1* gene, SOD1 protein, glucose, and protein carbonyl levels were positively correlated with the high-RFI groups on the right-hand side. In contrast, the PGAM1 and *TPI1* genes and the LDHB protein were located within the inner circle of the loading plot, indicating a limited contribution to the differentiation between low-RFI and high-RFI groups. Overall, the PCA results were largely consistent with the t-test findings presented in Figs.1–Fig. 2, Fig. 3, indicating that oxidative stress and metabolic regulation—particularly the expression of *SOD1* and *LDHB* genes, SOD1 protein abundance, and levels of glucose and protein carbonyl—play critical roles in determining FE in chickens. However, we noted a discrepancy in the case of LDHB; while the t-test revealed significant differences in protein abundance, its contribution appeared weak in PCA.

## Discussion

The results of growth performance in this study supported our initial hypothesis that feed intake (FI) significantly differs between low-RFI and high-RFI Korat chickens. This finding is consistent with previous studies by Liu et al. (2019), Poompramun et al. (2021), Sinpru et al. (2021), and Kaewsatuan et al. (2022), who reported that high-FE broilers and Korat chickens, respectively, consumed less feed than the low-FE groups, without significant differences in BWG. Kennedy et al. (1993) reported that several intrinsic factors—such as digestive capacity, energy requirements for maintenance and growth, and energy storage efficiency—likely contribute to these differences in energy balance. Thus, increased FI observed in high-RFI groups may be due to a greater reliance on dietary carbohydrates, proteins, and fats to meet elevated energy demands (Casal et al., 2020). These findings support the theoretical basis of RFI, where the goal of selection is not necessarily to maximize growth but rather to identify birds that utilize nutrients more efficiently at a given level of production (Kennedy et al., 1993; Wen et al., 2018; Sinpru et al., 2021). Hence, RFI was considered a more suitable trait for selection in this study.

Efficient energy metabolism is essential for FE in poultry and is strongly influenced by the duodenum, the primary site of nutrient digestion and absorption (Recoules et al., 2029; Yi et al., 2015). This intestinal segment plays a critical role in energy homeostasis by supporting ATP production through glycolysis and pyruvate metabolism—two interrelated pathways that facilitate glucose catabolism and mitochondrial respiration (Iqbal et al., 2005; Abasht et al., 2016). The results of the present study revealed the significant role of glycolytic genes in determining FE in slow-growing chickens. We found that chickens in the low-RFI groups exhibited significantly higher mRNA expression of triphosphate isomerase (*TPI1*) than the high-RFI groups. Also, the higher level of lactate dehydrogenase (*LDHB*) gene and protein expression was observed in the low-RFI groups compared to the high-RFI groups, consistent with previous studies (Rehman et al., 2018). *TPI1*, a key player in aerobic glycolysis, encodes a critical glycolytic enzyme that catalyzes the reversible interconversion of dihydroxyacetone phosphate (DHAP) and glyceraldehyde-3-phosphate (G3P) (Liu et al., 2022a). A well-regulated *TPI1* could ensure triose phosphate molecules derived from glucose are funneled into the energy-yielding phase, facilitating continuous flux of intermediates and optimizing the complete catabolism of glucose into pyruvate (Shah et al., 2019; Liu et al., 2022b; Myers et al., 2023). In addition, *LDHB* facilitates the conversion of lactate to pyruvate (Tavakoli et al., 2017). It is well known that pyruvate serves as the final product of glycolysis and a crucial substrate for ATP generation within mitochondria. The higher expression of the *TPI1* gene and *LDHB* gene and protein activity indicates that low-RFI chickens could be able to synthesize more pyruvate at a higher rate than high-RFI groups. Supporting this assumption, significant differences in glucose and pyruvate levels were also observed between low-RFI and high-RFI groups in the present study. Chickens in low-RFI groups exhibited lower plasma glucose concentrations and higher pyruvate levels compared to the high-RFI groups, indicating an increased metabolic flux from glucose and lactate toward pyruvate production. Previous studies have shown that heat-stressed birds exhibited a metabolically inflexible state in which glucose is preferentially utilized as the primary fuel for ATP production to meet their elevated energy demand (Liu et al., 2023). Collectively, our findings are consistent with previous studies and support the assumption that the high-FE animals require greater glucose uptake to generate ATP for protein accumulation and for maintaining tissue homeostasis (Dorji et al., 2021; Karimi et al., 2022), resulting in more efficient energy utilization observed in low-RFI groups.

Furthermore, it has been reported that mitochondrial inefficiency and oxidative stress are closely related to FE variation (Bottje and Cartens, 2008; Murphy, 2008). Although the mitochondrial respiration process is more efficient at producing ATP than glycolysis, it also generates reactive oxygen species (ROS) as an intrinsic byproduct—molecules, causing further oxidative stress by oxidizing proteins and DNA (Zelko et al., 2002; Miao et al., 2009). Several studies have reported that low-efficiency chickens tend to produce more mitochondrial ROS, which is associated with reduced efficiency of electron coupling in the mitochondrial electron transport chain (ETC) (Iqbal et al., 2004; Bottje et al., 2006). Although ROS production levels were not directly measured in the present study, our recent research demonstrated that high-RFI chickens exhibited higher mitochondrial ROS levels isolated from the duodenum compared to low-RFI chickens (Pengsanthia et al., 2025), supporting the assumption that inefficient birds may experience greater oxidative stress. In line with this, the present results showed that the antioxidant, i.e., superoxide dismutase1 (*SOD1*), was more highly expressed in the high-RFI groups compared to the low-RFI groups at both mRNA and protein levels. SOD1 is an important cellular antioxidant defense against oxidative stress in the body and serves to scavenge superoxide anions (O₂⁻) (Zhao et al. 2023). Increased SOD1 expression may therefore represent a compensatory response to elevated superoxide production, as has been observed in mitochondrial duodenal tissue (Shen et al., 2023) and breast muscle (Gonos et al., 2018) of low-FE broilers. In addition, protein carbonyl content is widely recognized as an indicator of oxidative stress and is by far the most commonly used marker for assessing oxidative damage to proteins (Iqbal et al., 2004). Consistent with our findings, accumulation of protein carbonyl content was observed in breast samples of lowly efficient chickens when compared to highly efficient chickens (Roy and Jacobson, 2013; Surai, 2016). The antioxidant response indicates that oxidative stress substantially increases the animal’s energy demands, thereby reducing the energy available for protein synthesis and ultimately lowering FE. Overall, we propose that chickens in the high-RFI group, which exhibit the highest levels of protein oxidation and *SOD1* gene expression, are likely experiencing oxidative stress and require a more efficient antioxidant defense system, which could impact their overall energy utilization efficiency.

According to our previous proteomic analysis (Kaewsatuan et al., 2022), duodenal proteins related to glycolysis (TPI1, ALDOC, LDHB, and PGAM1) and oxidative stress (SOD1 and PRDX1) were identified as potential biomarker candidates for FE regulation in slow-growing chickens. Their critical roles in maintaining cellular energy balance and influencing susceptibility to oxidative stress made them particularly interesting. However, in the current study, some glycolytic genes, specifically aldolase C (*ALDOC*) and phosphoglycerate mutase 1 (*PGAM1*), did not show the expected differential mRNA expression between RFI groups. This suggests that the previously observed changes in ALDOC and PGAM1 protein abundance and activity between RFI groups are likely not driven by transcriptional changes. This inconsistency between our proteomic and transcriptomic data for PGAM1 and ALDOC can be explained by multiple layers of regulatory complexity. This aligns with findings from other studies on intestinal energy metabolism (Zhang et al., 2022) and is supported by evidence that key glycolytic enzymes are often regulated via post-translational modifications (PTMs), such as phosphorylation, acetylation, and redox-based changes (Fernie et al., 2004). Hallows et al. (2012) provided the mechanistic evidence that PGAM1 interacts with sirtuin protein (SIRT1), demonstrating that dynamic acetylation–deacetylation cycles, especially under glucose restriction, regulate its glycolytic flux. Similarly, acetylation of ALDOC on lysine 42 (K42) can enhance its activity and promote glycolysis (Gao et al., 2022). These modifications can influence enzymatic activity independently of gene expression. Furthermore, PGAM1 and ALDOC are not rate-limiting enzymes in glycolysis (Chang and Hsiao, 2022). Because both enzymes catalyze reversible reactions with near-equilibrium free energy changes, their activities are primarily dependent on substrate and product concentrations, rather than tight allosteric or hormonal control (Yang et al., 2022). This inherent characteristic could explain their stable mRNA expression levels, likely reflecting a constitutive role in maintaining basal glycolytic function regardless of varying cellular energy demands between RFI groups (Fernie et al., 2004). Therefore, the observed proteomic differences might reflect adjustments at the protein level to fine-tune glycolytic capacity without necessarily requiring changes in gene transcription.

Additionally, we found no significant difference in the expression of peroxiredoxin 1 (*PRDX1*) between groups. The *PRDX1* gene encodes an enzyme involved in peroxisomal antioxidant metabolism and redox signaling. This observation is consistent with previous findings by Dorji et al. (2021), which suggested that peroxisomal antioxidant enzymes tend to remain relatively stable under normal metabolic conditions. Thus, it could be assumed that *PRDX1* might not be a primary player in redox adaptation when the chickens are not under major stress. In contrast to mammalian studies that reported PRDX1 upregulation in response to dietary or metabolic stress (Kim et al., 2018; Meghnem et al., 2022), our results suggest a different regulatory role in chickens. In avian species, mitochondrial antioxidant defenses appear to be more critical for maintaining redox balance than peroxisomal pathways (Bottje et al., 2006; Dunislawska et al., 2023; Pengsanthia et al., 2025). This indicates that the contribution of *PRDX1* to oxidative regulation in the duodenum may be limited under normal conditions, with mitochondrial antioxidant systems primarily responsible for adapting to variations in FE, playing a more significant role than peroxisomal pathways. Further studies incorporating direct measurements of mitochondrial function and ROS production are still needed to clarify the systemic mechanisms underlying redox regulation in relation to FE.

The Principal Component Analysis (PCA) provides compelling evidence for the clear separation of chicken groups based on their RFI values. This analysis further highlights specific genes, proteins, and metabolic molecules that most significantly contribute to FE variation, identifying them as potential informative biomarkers. As an unsupervised multivariate dimensionality reduction technique, PCA is a well-established method in omics research for exploring complex biological datasets (Misra et al., 2019; Torres-Espín et al., 2021). The PCA results revealed that the high-RFI and low-RFI groups formed two distinct clusters, underscoring clear biological differences among them. High-RFI chickens were characterized by strong positive loadings for RFI, blood glucose, SOD1 gene and protein expression, and protein carbonyl content, whereas LDHB gene expression showed a negative association with RFI. The coordinated reduction in *LDHB*-mediated energy flux, together with the elevated *SOD1*-driven antioxidant demand, suggests that high-RFI chickens may experience metabolic inflexibility, relying on less efficient energy pathways while incurring higher energetic costs to maintain redox balance. This interpretation aligns with previous findings that inefficient (high-RFI) chickens experience elevated cellular energy demands to maintain redox homeostasis in response to increased oxidative stress, along with reduced glycolytic efficiency, ultimately lowering feed efficiency (Bottje et al., 2006; Ye et al., 2025). Taken together, the integration of multi-omics approaches (qPCR, Western blot, metabolic assays), and multivariate analysis (PCA) in the present study provides strong evidence that *LDHB* and *SOD1* function as direct molecular markers of feed efficiency in chickens. Specifically, reduced *LDHB* expression in high-RFI birds corresponded with decreased pyruvate flux into the TCA cycle, thereby limiting mitochondrial ATP production. In contrast, elevated *SOD1* expression in the high-RFI birds was associated with increased antioxidant demand, reflecting higher level of protein oxidative damage in the duodenal tissue and imposing additional energetic costs. Thus, these findings bridge associative correlations with mechanistic insight by linking altered *LDHB* and *SOD1* expression to the regulation of cellular energy metabolism, supporting their role as direct contributors to FE variation.

However, several limitations of the present work should be acknowledged. First, although the LDHB protein was statistically significant in differential analysis, it exhibited weak loading in the PCA. This likely reflects the fact that PCA emphasizes variables explaining the largest proportion of total variance, whereas LDHB, despite being biologically relevant, contributed relatively little to the multivariate separation. Second, although the duodenum was selected as the focal tissue due to its central role in nutrient absorption, FE is inherently a systemic trait arising from the coordinated activity of multiple organs, including the liver (Karimi et al., 2022) and skeletal muscle (Omotoso et al., 2024). Therefore, further studies incorporating multi-tissue analyses will be necessary to provide a more comprehensive understanding of the molecular networks that govern FE.

## Conclusion

Overall, our findings significantly advance the understanding of the molecular mechanisms underlying RFI, particularly those related to glycolysis and oxidative stress in the chicken duodenum, and highlight potential molecular targets for improving FE. Our results indicate that both the glycolytic rate and the management of oxidative stress within the duodenum critically impact energy utilization in chickens. Specifically, efficient chickens (low-RFI) tend to demonstrate a greater capacity for energy generation and more efficient energy partitioning towards growth compared to inefficient ones (high-RFI). Through systematic analysis, *LDHB* and *SOD1* emerge as potential core molecular markers associated with variations in RFI in chickens. Our study paved the way for future investigations into FE regulation and advancing genetic selection strategies in chickens.

## CRediT authorship contribution statement

**Rattanaporn Niyomphong:** Writing – review & editing, Writing – original draft, Visualization, Validation, Investigation, Data curation. **Pramin Kaewsatuan:** Writing – review & editing, Visualization, Investigation. **Saknarin Pengsanthia:** Validation, Software, Writing – review & editing. **Wittawat Molee:** Methodology, Conceptualization, Writing – review & editing. **Amonrat Molee:** Writing – review & editing, Supervision, Project administration, Methodology, Conceptualization.

## Disclosures

The authors declare that they have no known competing financial interests or personal relationships that could have appeared to influence the work reported in this paper.
